# The Influence of Obesity on Melanoma and Sentinel Lymph Node Diagnosis: A Retrospective Monocentric Study in 1001 Patients

**DOI:** 10.3390/cancers15061806

**Published:** 2023-03-16

**Authors:** Filipa Almeida Oliveira, Julie Klose, Hans-Joachim Schulze, Marta Ribeiro Teixeira, Alexander Dermietzel, Sascha Wellenbrock, Grit-Sophie Herter-Sprie, Tobias Hirsch, Maximilian Kueckelhaus

**Affiliations:** 1 Department of Plastic and Reconstructive Surgery, Institute of Musculoskeletal Medicine, University Hospital Muenster, 48149 Muenster, Germany; 2 Department of Plastic, Reconstructive and Aesthetic Surgery, Hand Surgery, Fachklinik Hornheide, 48157 Muenster, Germany; 3 Division of Plastic Surgery, Department of Trauma, Hand and Reconstructive Surgery, University Hospital Muenster, 48149 Muenster, Germany; 4 Department of Dermatology, Dermatological Radiotherapy and Dermatohistopathology, Fachklinik Hornheide, 48157 Muenster, Germany; 5 Dermatology Group Practice, 48145 Muenster, Germany; 6 Department of Oncology, Fachklinik Hornheide, 48157 Muenster, Germany; 7 Department I of Internal Medicine, Faculty of Medicine, University Hospital Cologne, University of Cologne, 50935 Cologne, Germany

**Keywords:** melanoma, obesity, body mass index, Breslow thickness, sentinel lymph node, sentinel lymph node biopsy, metastasis

## Abstract

**Simple Summary:**

The impact of obesity on melanoma has rarely been researched. Incidence of obesity is rapidly growing and melanoma is one of the most prevalent types of cancers worldwide. Several studies have shown that overweight and obese populations not only have a higher risk of developing melanoma but also tend to present with thicker melanomas at the time of diagnosis. Given that melanoma thickness is one of the main predictors of the melanoma prognosis, a worse prognosis in a patient with obesity would be expectable. However, this has not yet been demonstrated in the literature. Our study is the first to show that obese patients are twice more likely to present with lymph-node metastases. Lymph node metastases is the second most important prognosis predictor of melanoma. Our findings, therefore, raise important questions regarding the screening and treatment of obese patients with melanoma.

**Abstract:**

(1) Background: While obesity is a known independent risk factor in the development of melanoma, there is no consensus on its influence on melanoma prognosis. (2) Methods: In a monocentric retrospective study, data was collected from patients who underwent sentinel lymph node (SLN) biopsy for stage IB-IIC melanoma between 2013 and 2018. Patients were divided into groups according to their body mass index (BMI). The association between BMI and melanoma features, as well as the risk factors for metastases in SLN were examined. (3) Results: Of the 1001 patients, 336 had normal weight (BMI < 25), 402 were overweight (BMI >= 25 and <30), 173 obese (BMI >= 30 and <35) and 90 extremely obese (BMI >= 35). Overweightness and obesity were associated with higher tumor thicknesses at time of diagnosis. Ulceration was not influenced by the patient’s weight. Metastases in sentinel lymph node was almost twice more likely in extremely obese patients than in normal weight patients. Independent risk factors for metastases in SLN in our study were tumor thickness, ulceration, and BMI > 35. (4) Conclusions: This is the first study to show higher metastases rates in high-BMI patients with melanoma, raising important questions regarding the screening and treatment of this specific patient population.

## 1. Introduction

Melanoma is one of the most common malign tumors worldwide and its incidence is increasing [1,2]. The risk of melanoma development depends on an interaction between environmental factors, most importantly UV radiation, and predisposing host factors. The latter consist of genetic predisposition, phenotype, family history, and number of melanocytic nevi [2].

Obese populations have been associated with a higher risk of developing several cancers, including melanoma. This was shown by Oh et al., who prospectively analyzed the risk of cancer development in a cohort of over 700,000 healthy men in Korea over a 10-year period [3] and by Samanic et al. in a cohort of 4,500,700 male American veterans, over a period of 27 years [4]. Similar results linking obesity to melanoma development were found in a prospective Scandinavian study with 362,552 Swedish men [5]. Dennis et al. also found a clear association between melanoma development and obesity in a prospective cohort of farmers and their spouses, who were followed up for 10 years [6]. A correlation between melanoma and obesity was also found in two case-control studies, in which obesity prevalence in melanoma patients was higher than in the control groups [7,8].

Obesity not only seems to predispose cancer development but also to negatively influence the prognosis of several cancer entities such as colorectal, liver, gallbladder, pancreatic, breast, and ovarian cancers, among others [9]. Whether obesity also negatively influences melanoma prognosis has been studied by several authors [10].

Melanoma prognosis at time of diagnosis is defined by three main tumor features: Breslow tumor thicknesses, ulceration, and metastases in sentinel lymph nodes (SLN) [11].

The association between Breslow tumor thickness and obesity is not unanimous. Skowron et al. studied 427 melanoma patients and concluded that BMI >= 30 was an independent risk factor for the development of thick melanoma [12]. Similar results were found by Gandini et al. in an multicentric Italian study with 2738 patients. In this study, this association could already be observed at BMI >= 25 [13]. Other authors described a gender-based relation between obesity and melanoma. Giorgi et al. found an association between melanoma with tumor thickness >= 1 mm and BMI >= 25 only in females, especially postmenopausal [14], whereas Stenehjem et al., with the largest cohort of 2570 patients, only discovered an association between tumor thickness and higher BMI in males [15].

Ulceration has been investigated for its relation to obesity, but no significant correlation was found [12,16,17].

Metastases in sentinel lymph nodes and their relationship with obesity has only been mentioned by Shreckengost et al. as not existent [18]. To our knowledge, no previous studies have addressed this possible causality.

The aim of our study was to understand the influence of obesity on melanoma features and melanoma main predictors. The study was performed in a high-volume center for skin malignancy care.

## 2. Materials and Methods

In our institution, stage IB-IIC melanoma patients received a sentinel lymph node biopsy in the Department of Plastic Surgery or in the Department of Oral-Maxillofacial Surgery, based on the respective tumor location.

### 2.1. Data Collection

Retrospectively, patients were identified as those with melanoma Stage IB-IIC in the trunk and limbs and who were submitted to sentinel lymph node biopsy between 2013 and 2018 at our institution.

Patient data (age, gender, and body mass index—BMI) and tumor characteristics (tumor location, Breslow tumor thickness, ulceration, metastases in sentinel lymph node, extracapsular spread, S100 value) were extracted from the hospital’s internal information system. Inclusion criteria were age over 18 years old, known body mass index and known Breslow tumor thickness.

### 2.2. Data Analysis

Patients were divided in four groups according to their BMI: normal weight (BMI >= 18.5 and <25), overweight (BMI >= 25 and <30), obese (BMI >= 30 and <35) and extremely obese (BMI >= 35). Differences between BMI groups with respect to age, gender, and melanoma characteristics were analyzed. To analyze the overall differences between the BMI groups, Fisher’s test and Chi-square test were used for categorical characteristics (Table 1), and Kruskal–Wallis test was used for metric characteristics (Table 2). If the *p* value of these global tests was less than the selected significance level (*p* < 0.5), then the BMI groups were compared in pairs with the Mann–Whitney U test and the significance level was adjusted with the Bonferroni–Holm procedure to find significant differences between BMI groups (Table 3 and Table 4).

Additionally, each patient and tumor characteristic was analyzed for the chance of metastases in the sentinel lymph node biopsy using odds ratio with 95% confidence interval (OR (95% CI)) (Table 5 and Table 6). A sub-analysis of the risk of metastases in the sentinel lymph node biopsy was performed on patients with TD < 1 mm.

Furthermore, two multivariate analyses (logistic regressions) were calculated, one where all characteristics were considered (full model) and one where only characteristics with significant influence on sentinel lymph node positivity (*p* < 0.05) were taken into account (stepwise selection) (Table 7).

## 3. Results

### 3.1. Demographic Characteristics

Patients whose tumor thickness was unknown or not accurately determined (n = 10) were excluded. A total of 1001 patients met the inclusion criteria for our study.

The median age was 58.0 ± SD 15.3 years and the median Breslow tumor thickness was 2.9 ± SD 4.1 mm. Trunk was the most frequent tumor location (415–41.5%), followed by the lower extremity (363–36%) and the upper extremity (223–22%). Twenty-two percent of the patients had ulcerated melanomas. Of 1001 patients, 483 (48.3%) were male and 518 (51.7%) were female. A total of 336 (34%) patients had normal weight (BMI >= 18.5 and <25), 402 (40%) were overweight (BMI >= 25 and <30), 173 (17%) were obese (BMI >= 30 and <35) and 90 (9%) extremely obese (BMI >= 35). Underweight patients (BMI < 18.5) were excluded from our study. A total of 37.9% of patients had metastases in the sentinel lymph node biopsy and 10.0% of the patients presented with extracapsular spread.

### 3.2. Analysis of BMI Groups

BMI groups statistically differed regarding Breslow tumor thickness, metastases in sentinel lymph node biopsy, age, and gender.

**Figure 1:**
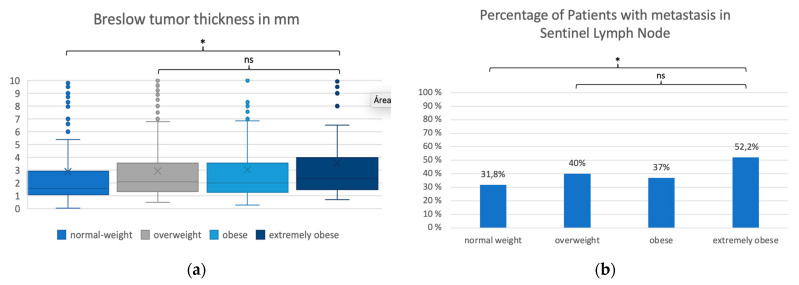
Comparison of BMI groups regarding Breslow tumor thickness (**a**) and metastases in sentinel lymph node (**b**). In (**a**), the y axis represents Breslow tumor thickness, and the x axis represents the different BMI groups. BMI groups: normal weight (BMI < 25), overweight (BMI >= 25 and <30), obese (BMI >= 30 and <35) and extremely obese (BMI >= 35). Breslow Tumor thickness was lower in normal weight patients (median 1.6 ± SD 5.9 mm) than in all patients with BMI >= 25 (respectively, 2.1 ± SD 2.5 mm, 2.0 ± SD 3.2 mm and 2.3 ± SD 3.2 mm) (* *p* < 0.001, *p*-Value Kruskal–Wallis test). Tumor thickness did not differ statically between the overweight and obese groups. In (**b**), the y axis represents percentage of patients in each group, and the x axis represents the different BMI groups. Sentinel lymph node was positive in 31.8% of normal-weight patients compared to 52.2% of extremely obese patients. (* *p* < 0.001, *p*-value Fisher’s test). ns = statistically non-significant.

Breslow tumor thickness in the normal-weight patients (median 1.6 ± SD 5.9 mm) was lower than in all the other BMI groups (*p* < 0.002, *p*-Value Kruskal–Wallis test). Among overweight, obese, and extremely obese groups, differences in Breslow tumor thickness were not statistically significant (respectively, 2.1 ± SD 2.5 mm, 2.0 ± SD 3.2 mm and 2.3 ± SD 3.2 mm) (*p* > 0.1) (Table 1 and Table 3 and Figure 1a).

Normal-weight patients were younger (median age 54 ± SD 16.9 years) than overweight patients (median 60.0 ± SD 14.6 years) and obese patients (median 60.0 ± SD 13.2 years) (*p* < 0.001). Extremely obese patients (median 58 ± SD 12.5) did not statistically differ from any other group regarding age (Table 1 and Table 3).

The number of women was statically higher in the normal weight (66.1%) and in the extremely obese groups (62%) than in the overweight and obese groups (respectively, 40.0% and 45.7%, *p* < 0.001 and *p* = 0.013) (Table 2 and Table 4).

Extracapsular spread, tumor location and ulceration did not statistically differ between the BMI groups (Table 2).

BMI groups differed regarding the percentage of patients with metastases in the sentinel lymph node biopsy, which increased with the BMI. A statistically significant difference between the groups could only be observed between the extremely obese BMI group (52.2%) and the normal weight group (31.8%) (*p* = 0.001) (Table 2 and Figure 1b).

### 3.3. Metastases in the Sentinel Lymph Node Biopsy

Our analysis showed that the higher the BMI (OR 1.04, CI 1.01–1.06), age (OR 1.01, CI 1.00–1.02), Breslow tumor thickness (OR 1.11, CI 1.07–1.14), and S100 value (OR 1.45, CI 0.39–5.45), the higher the likelihood of the patient exhibiting metastases in the sentinel lymph node biopsy (respectively, *p* = 0.005, *p* = 0.057, *p* < 0.001, *p* = 0.0105, Table 5).

Furthermore, extremely obese patients (BMI >= 35) were almost twice more likely (OR 1.91, CI 1.23–2.95) to have a metastatic sentinel lymph node than normal-weight patients (*p* = 0.004, Table 6).

In the sub-analysis of the patients with Breslow tumor thickness under 1 mm (n = 122), extremely obese patients (BMI >= 35) were four times more likely (OR 4.27, CI 0.78–23.31) to have a metastatic sentinel lymph node than normal-weight patients (*p* = 0.100, Table 8).

In the initial multivariate analysis, the chance of having a positive sentinel lymph node in our population was higher being a male (OR 0.79, CI 0.58–1.08, *p* = 0.142), being overweight or extremely obese (OR 1.20, CI 0.85–1.70, *p* = 0.299 and OR 2.10, CI 1.30–3.65, *p* = 0.009). Melanomas on trunk or lower extremity were more likely to have metastases in the sentinel lymph node biopsy (OR 1.52, CI 1.04–2.23, *p* = 0.032 and OR 1.36, CI 0.91–2.04, *p* = 0.131). Ulceration and Breslow tumor thickness were also associated with metastases in the sentinel lymph node biopsy (OR 1.76, CI 1.16–2.67, *p* = 0.008 and OR 1.18, CI 1.00–1.40, *p* = 0.050).

In the stepwise selection, where only characteristics with a significant influence on sentinel lymph node positivity (*p* < 0.05) were taken into consideration, only an association between metastases in sentinel lymph node biopsy and BMI >= 35 (OR 1.99, CI 1.15–3.42, *p* = 0.013), Breslow tumor thickness (OR 1.79, CI 1.18–2.70, *p* = 0.006), and Ulceration (OR 1.18, CI 1.01–1.38, *p* = 0.040) could be shown. For the other characteristics, such as male gender, tumor location and overweight, the odds ratio was no longer significantly different from 1.

## 4. Discussion

This study demonstrates a significantly higher tumor Breslow thickness in overweight and obese melanoma patients. Extreme obesity was identified as an independent risk factor for the presence of lymph-node metastases.

In our study, only 34% of the patients who received a sentinel lymph node biopsy between 2013 and 2018 had a normal body weight (BMI < 25). Most patients (66%) were overweight, obese, or extremely obese (BMI >= 25). The prevalence of obesity and overweightness in the studied population was higher than in the general German population (54%) [19]. These findings corroborate the results of other authors who show an increased risk of melanoma development in overweight and obese populations [3,4,6,7,8].

Furthermore, in our study, at time of diagnosis, overweight, obese, or extremely obese patients presented with higher tumor thicknesses than normal-weight patients. These results are in-line with findings in previous research that overweightness and obesity are associated with thicker melanomas [10,12,13]. Several theories have attempted to explain the relation between obesity and melanoma development and progression. The metabolic role of obesity in tumor growth has been well-documented for several tumor entities. Obesity and its chronic calory excess lead to abnormal levels of glycemia, insulin, cytokines, adipokines and steroid hormones. This, in turn, leads to a pro-inflammatory state and promotes tumor progression and angiogenesis [20]. The same mechanisms may also induce melanoma growth in overweight and obese patients [21,22]. In addition to the metabolic activity from adipose tissue, diet has been proposed to also influence tumor development. Preclinical studies have shown that a high-fat diet may induce melanoma progression [23] and, on the other hand, that caloric restriction may slow down melanoma growth [21]. In a clinical study from Norway with 50,752 participants, a diet rich in omega-3 fatty acids and in polyunsaturated fat was associated with an increased melanoma risk in women [24]. Some authors have searched for a genetic mutation conferring simultaneous susceptibility to melanoma and obesity [25]. Cauci et al. focused on polymorphisms from a vitamin-D receptor [26]. Li et al. studied single nucleotide polymorphisms in the FTO, MAP2K5, NEGR1, FLJ35779, ETV5, CADM2, and NUDT3 genes [27]. These hypotheses remain to be confirmed.

The literature also refers to the relation between tumor progression and late tumor detection due to hidden tumor location. This theory was refuted by Skowron et al., who showed that melanomas in overweight or obese patients are not more frequent in non-visible body areas than in normal-weight patients [12]. Late tumor detection and melanoma progression due to avoidance of doctor appointments possibly because of lower self-esteem in overweight and obese populations was studied by Risica et al. The authors showed that the method of melanoma detection, through self-examination or doctor appointments, does not seem to differ with BMI [28].

In our results, although Breslow tumor thickness was higher in all overweight and obese patients (with a BMI >= 25), Breslow tumor thickness did not linearly increase with BMI, meaning it did not differ within overweight, obese, and extremely obese patients. Therefore, our study shows that there is a higher risk of melanoma progression in patients with a BMI >= 25, but this risk does not continue to increase from BMI >= 25 and is the same for overweight, obese, and extremely obese patients.

Ulceration and S100 value do not seem to be influenced by overweight or obesity, since they did not differ within BMI groups in our study. These findings are in-line with other groups [12,15].

A higher rate of non-detection of the sentinel lymph node despite lymphoscintigraphy has been described for breast cancer in obese patients [29,30]. Similar studies regarding sentinel lymph node biopsy in melanoma could not be found. In our study, the non-detection of SLN was not documented.

There is little research on the role of obesity in the development of melanoma metastases. To our knowledge, only Shreckengost et al. analyzed the impact of obesity on sentinel lymph node metastases [18]. Contrary to their results, our study showed a significant influence of BMI >= 35 on sentinel lymph node metastases, one of the main predictors of melanoma prognosis. However, our research differs from Shreckengost et al.’s study with regards to the study design. Patients with melanoma in stage IA were included in Shreckengost et al.’s research, while they were not included in ours. Moreover, in 43.1% of patients, SLN data was missing in Shreckengost et al.’s study. Thus, overall metastases in the sentinel lymph node biopsy in the aforementioned study was only found in 15.1% of the patients in contrast to our population, where 37.9% of the patients exhibited metastases in the sentinel lymph node biopsy. Furthermore, patient groups in Shreckengost et al.’s study and in this study differed as we distinguished patients with a BMI >= 35.

Moreover, in our study, extremely obese patients (BMI >= 35) were almost twice more likely (OR 1.99) to have metastases in the sentinel lymph node than normal-weight patients. Extracapsular spread, however, did not differ within both groups. In the initial multivariate analysis, male gender, tumor location on body trunk or lower extremity as well as advanced age seemed to be predictors of metastases in the sentinel lymph node. After the stepwise selection, only BMI >= 35, Breslow tumor thickness, and ulceration were independent predictors of metastases in sentinel lymph node in our population. Since the last two tumor characteristics are the most important and validated features of melanoma prognosis and, therefore, of melanoma survival [11], our findings regarding BMI >= 35 and metastases in the sentinel lymph node may be valid.

BMI is a standardized and inexpensive method to assess obesity, but it does not consider muscle mass or differentiate visceral from subcutaneous fat [31]. Other assessment methods of obesity such as hip circumference, waist-to-height, and waist-to-hip ratio also fail to measure visceral fat [32]. Among others, air displacement plethysmography [33], dual-energy X-ray and magnetic resonance imaging [34] are very accurate assessments of visceral fat but are undoubtedly more expensive and difficult to reproduce in such a big population study as ours. Despite its limitations, the authors consider that patients with a BMI >= 35 unquestionably suffer from morbid obesity.

The mechanisms leading to the higher frequency of metastases presence in patients with BMI >= 35 are not yet identified. The main differentiating feature of the extremely obese patients’ group is that of having the highest amount of adipose tissue in comparison to all other BMI groups.

Adipose tissue, besides the aforementioned endocrine and metabolic activities, is known to cause a systemic immunological disfunction and a reduced response to cancer in obese patients [35,36]. Some authors have observed, contrary to expectation, that obese patients with metastatic melanoma seem to have a better therapeutic response than normal-weight patients, especially to immunotherapy [37,38,39]. This is referred to as the “obesity paradox” [35,37]. Wang et al. showed that the immunological function of T cells was altered in obese patients (BMI >= 30) and the expression of PD-1 was higher, making a better efficacy of autoimmune therapy possible [35].

Our findings raise at least two questions regarding the screening and treatment of overweight and obese patients: Firstly, if overweight and obese populations are more likely to develop melanomas and they present with higher tumor thicknesses, should skin-cancer screening be adjusted to patient’s weight? Skin-cancer screening in Germany is recommended from the age of 35 years and is paid for by the public medical insurance every 2 years [40]. An earlier and/or more frequent screening could lead to an earlier discovery of melanoma with lower tumor thickness and, consequently, to a better prognosis of these patients. Secondly, if extremely obese patients (BMI >= 35) are more likely to exhibit metastases in the sentinel lymph node (although tumor thickness does not differ from overweight or obese patients), should BMI >= 35 also be a criterion to offer sentinel-node surgery in patients with lower tumor thicknesses (<1.0 mm)? Sentinel lymph node surgery is offered to all patients with tumor Breslow thickness over 1.0 mm. An earlier sentinel lymph node surgery is also indicated in patients with tumor thicknesses >0.75 mm and <1.0 mm, who meet certain criteria such as ulceration, increased mitosis rate and age under 40 years [41]. An earlier SLN surgery in patients with BMI >= 35 could lead to an earlier detection of metastases and, therefore, improve the treatment and prognosis of these patients. Following this line of thought, we analyzed whether the same results could be found in patients with tumor thicknesses <1.0 mm by conducting the same statistical analysis of our study but restricted to these patients (Table 8 and Table 9). In this population group, a similar trend regarding metastases in the sentinel lymph node was identified. However, the differences did not reach statistical significance, possibly due to the small number of patients. A multicenter study with a higher number of patients may help explain whether extremely obese patients would benefit from these approaches, which may eventually lead to a necessity of adjustment of the melanoma treatment guidelines.

Limitations of our study are the absence of head and neck melanomas, its retrospective nature, and its single-center approach.

## 5. Conclusions

In conclusion, our study not only validated that BMI influences melanoma development but also affects two of the main melanoma predictors: Breslow tumor thickness and metastases in the sentinel lymph node. BMI >= 25 was associated with thicker melanomas at diagnosis and BMI >= 35 was associated with an almost twice higher likelihood of exhibiting metastases in the sentinel lymph node than normal patients. Therefore, patients with a BMI >= 25 and, in particular, patients with BMI >= 35 had a worse prognosis at the time of diagnosis. To our knowledge, this is the first study to show this association.

Our study raises the question of whether the prognosis of obese patients can be optimized through earlier tumor detection or through sentinel-node surgery at lower tumor thicknesses.

## Figures and Tables

**Table 1:** Description and comparison of BMI groups—metric characteristics.

	BMI Group	n	Mw	SD	Median	Min-Max	*p*-Value * (Global Test)
Age	Normal weight (>=18.5 and <25)	336	53.8	16.9	54.0	22.0–92.0	
	Overweight (>=25 and <30)	402	60.0	14.6	60.0	21.0–95.0	
	Obese (>=30 and <35)	173	60.0	13.2	60.0	24.0–89.0	
	Extremely obese (>= 35)	90	57.8	12.5	58.0	30.0–81.0	
	Total	1001	57.7	15.3	58.0	21.0–95.0	<0.001
Breslow	Normal weight (>=18.5 and <25)	336	2.9	5.9	1.6	0.04–75.0	
tumor	Overweight (>=25 and <30)	402	2.9	2.5	2.1	0.5–17.0	
thickness	Obese (>=30 and <35)	173	3.0	3.2	2.0	0.3–32.0	
	Extremely obese (>=35)	90	3.5	3.2	2.3	0.7–14.2	
	Total	1001	3.0	4.1	1.9	0.04–75.0	<0.001
S100 value	Normal weight (>=18.5 and <25)	306	0.1	0.1	0.1	0.0–1.5	
	Overweight (>=25 and <30)	370	0.1	0.1	0.1	0.0–0.8	
	Obese (>=30 and <35)	159	0.1	0.1	0.1	0.0–0.8	
	Extremely obese (>= 5)	81	0.1	0.1	0.1	0.0–0.6	
	Total	916	0.1	0.1	0.1	0.0–1.5	0.139

* *p*-value Kruskal–Wallis test.

**Table 2:** Description and comparison of BMI groups—categorical characteristics.

		Patients (n = 1001)	Normal Weight (BMI >= 18.5 and <25)	Overweight (BMI >= 25 and <30)	Obese (BMI >= 30 and <35)	Extremely Obese (BMI >= 35)	*p*-Value * (Global Test)
Gender	Male	483 (48.3%)	114 (33.9%)	241 (60.0%)	94 (54.3%)	34 (37.8%)	
	Female	518 (51.7%)	222 (66.1%)	161 (40.0%)	79 (45.7%)	56 (62.2%)	*p* < 0.001
Sentinel	Negative	622 (62.1%)	229 (68.2%)	241 (60.0%)	109 (63.0%)	43 (47.8%)	
Lymph Node	Positive	379 (37.9%)	107 (31.8%)	161 (40.0%)	64 (37.0%)	47 (52.2%)	*p* = 0.003
Extracapsular	No	901 (90.0%)	306 (91.1%)	367 (91.3%)	149 (86.1%)	79 (87.8%)	
spread	Yes	100 (10.0%)	30 (8.9%)	35 (8.7%)	24 (13.9%)	11 (12.2%)	*p* = 0.204
Tumor	Body trunk	415 (41.5%)	140 (41.7%)	169 (42.0%)	76 (43.9%)	30 (33.3%)	
location	Upper extremity	223 (22.3%)	73 (21.7%)	85 (21.1%)	37 (21.4%)	28 (31.1%)	
	Lower extremity	363 (36.3%)	123 (36.6%)	148 (36.8%)	60 (34.7%)	32 (35.6%)	*p* = 0.495
Ulceration	No	780 (78.0%)	263 (78.3%)	312 (77.8%)	134 (77.5%)	71 (78.9%)	
	Yes	220 (22.0%)	73 (21.7%)	89 (22.2%)	39 (22.5%)	19 (21.1%)	*p* = 0.993

* *p*-Value Fisher–Test, with exception of location: *p*-value of the chi-square test.

**Table 3:** *p*-values of the pairwise comparisons of the BMI groups (Mann–Whitney U test).

			Age	Breslow Tumor Thickness	S100 Value
Normal weight	vs.	overweight	<0.001 #	<0.001 #	0.127
Normal weight	vs.	obese	<0.001 #	0.002 #	0.098
Normal weight	vs.	extremely obese	0.039	<0.001 #	0.057
Overweight	vs.	obese	0.800	0.823	0.563
Overweight	vs.	extremely obese	0.119	0.173	0.273
Obese	vs.	extremely obese	0.200	0.192	0.596

# significant according to Bonferroni–Holm adjustment for multiple testing, adjustment per characteristic.

**Table 4:** *p*-values of the pairwise comparisons (Fisher-test).

			Gender	Metastases in SLN	Extracapsular Spread	Location	Ulceration
Normal weight	vs.	overweight	<0.001 #	0.021	1.000	0.986	0.929
Normal weight	vs.	obese	<0.001 #	0.276	0.095	0.878	0.822
Normal weight	vs.	extremely obese	0.534	0.001 #	0.420	0.145	1.000
Overweight	vs.	obese	0.231	0.515	0.072	0.891	0.913
Overweight	vs.	extremely obese	<0.001 #	0.044	0.317	0.106	0.889
Obese	vs.	extremely obese	0.013 #	0.025	0.849	0.146	0.876

# Significant according to Bonferroni–Holm adjustment for multiple testing, adjustment per characteristic.

**Table 5:** Metastases in the sentinel lymph node biopsy—metric characteristics.

	SLN	N	Mw	SD	Median	Min-Max	OR (95%-CI)	*p*-Value
BMI	Negative	622	27.2	5.0	26.6	18.6–55.5		
	Positive	379	28.1	5.4	27.3	18.6–49.9	1.04 (1.01–1.06)	0.005
Age	Negative	622	57.0	15.2	58.0	21.0–89.0		
(Years)	Positive	379	58.9	15.3	60.0	21.0–95.0	1.01 (1.00–1.02)	0.057
Breslow tumor	Negative	622	2.3	3.5	1.6	0.04–75.0		
Thickness (mm)	Positive	379	4.1	4.9	2.9	0.2–70.0	1.11 (1.07–1.14)	<0.001
S100 value	Negative	573	0.1	0.1	0.1	0.0–1.4		
	Positive	343	0.1	0.1	0.1	0.0–1.5	1.45 (0.39–5.45)	0.105

**Table 6:** Metastases in the sentinel lymph node biopsy-categorical characteristics.

		n	SLN Negative	SLN Positive	OR (95%-CI)	*p*-Value
Gender	Male	483	283 (58.6%)	200 (41.4%)		
	Female	518	339 (65.4%)	179 (34.6%)	0.75 (0.58–0.97)	0.027
BMI	Normal weight (>=18.5 and <25)	336	229 (68.2%)	107 (31.8%)		
	Overweight (>=25 and <30)	402	241 (60.0%)	161 (40.0%)	1.43 (1.05–1.94)	0.021
	Obese (>=30 and <35)	173	109 (63.0%)	64 (37.0%)	1.03 (0.73–1.45)	0.861
	Extremely obese (>=35)	90	43 (47.8%)	47 (52.2%)	1.91 (1.23–2.95)	0.004
Tumor	Upper Extrem.	223	154 (69.1%)	69 (30.9%)		
location	Trunk	415	251 (60.5%)	164 (39.5%)	1.46 (1.03–2.06)	0.038
	Lower Extrem.	363	217 (59.8%)	146 (40.2%)	1.50 (1.05–2.14)	0.027
Ulceration	No	780	523 (67.1%)	257 (32.9%)		
	Yes	220	98 (44.5%)	122 (55.5%)	2.53 (1.86–3.46)	<0.001

**Table 7:** Metastases in the sentinel lymph node biopsy—multivariate analysis (logistic regression) (n = 1001).

		Full Model	Stepwise Selection
		OR (95%-CI), *p*-Value	OR (95%-CI), *p*-Value
Gender	Female vs. male	0.79 (0.58–1.08), *p* = 0.142	
BMI	>=18.5 and <25	Ref	Ref
	>=25 and <30	1.20 (0.85–1.70), *p* = 0.299	1.26 (0.90–1.77), *p* = 0.173
	>=30 and <35	1.09 (0.70–1.69), *p* = 0.698	1.12 (0.73–1.72), *p* = 0.610
	>=35	2.10 (1.20–3.65), *p* = 0.009	1.99 (1.15–3.42), *p* = 0.013
Location	Upper extremity	Ref	
	Trunk	1.52 (1.04–2.23), *p* = 0.032	
	Lower extremity	1.36 (0.91–2.04), *p* = 0.131	
	Ulceration (yes vs. no)	1.76 (1.16–2.67), *p* = 0.008	1.79 (1.18–2.70), *p* = 0.006
	Age (years)	1.00 (0.99–1.01), *p* = 0.619	
	Breslow tumor thickness (mm)	1.18 (1.00–1.40), *p* = 0.050	1.18 (1.01–1.38), *p* = 0.040

**Table 8:** Metastases in the sentinel lymph node biopsy in patients with Breslow tumor thickness <1 mm—categorical characteristics.

		Patients (n = 122)	SLN without Metastases	SLN with Metastases	OR (95%-KI)	*p*-Value
Gender	Male	46	36 (78.3%)	10 (21.7%)		
	Female	76	61 (80.3%)	15 (19.7%)	0.89 (0.36–2.19)	0.820
BMI	>=18.5 and <25	61	47 (77.0%)	14 (23.0%)		
	>=25 and <30	36	30 (83.3%)	6 (16.7%)	0.67 (0.23–1.96)	0.605
	>=30 and <35	19	17 (89.5%)	2 (10.5%)	0.45 (0.10–2.15)	0.522
	>=35	6	3 (50.0%)	3 (50.0%)	4.27 (0.78–23.31)	0.100
Tumor	Body trunk	19	15 (78.9%)	4 (21.1%)		
location	Upper extremity	54	41 (75.9%)	13 (24.1%)	1.19 (0.33–4.26)	1.000
	Lower extremity	49	41 (83.7%)	8 (16.3%)	0.73 (0.19–2.82)	0.727
Ulceration	No	115	92 (80.0%)	23 (20.0%)		
	Yes	7	5 (71.4%)	2 (28.6%)	1.60 (0.29–8.86)	0.631

**Table 9:** Metastases in the sentinel lymph node biopsy in patients with Breslow tumor thickness <1 mm—metric characteristics.

	SLN	n	Mw	SD	Median	Min-Max	OR (95%-CI)	*p*-Value
BMI	Negative	97	25.8	4.5	25.5	18.8–38.0		
	Positive	25	26.2	5.8	24.3	18.8–40.9	1.02 (0.93–1.11)	0.887
Age	Negative	97	49.5	15.1	50.0	21.0–85.0		
(Years)	Positive	25	50.2	13.2	50.0	25.0–77.0	1.00 (0.97–1.03)	0.884
Breslow tumor	Negative	97	0.8	0.2	0.8	0.04–1.0		
Thickness (mm)	Positive	25	0.8	0.2	0.9	0.2–1.0	0.97 (0.07–14.09)	0.379
S100 value	Negative	88	0.1	0.1	0.1	0.0–0.6		
	Positive	21	0.1	0.0	0.1	0.0–0.1	0.03 (0.00–26.07)	0.413

## Data Availability

The data can be shared upon request.
